# Particle Imaging Velocimetry Evaluation of Intracranial Stents in Sidewall Aneurysm: Hemodynamic Transition Related to the Stent Design

**DOI:** 10.1371/journal.pone.0113762

**Published:** 2014-12-03

**Authors:** Pierre Bouillot, Olivier Brina, Rafik Ouared, Karl-Olof Lovblad, Mohamed Farhat, Vitor Mendes Pereira

**Affiliations:** 1 Interventional Neuroradiology Unit, Service of Neuroradiology, University Hospital of Geneva, Genève, Switzerland; 2 Laboratory for Hydraulic Machines, École Polytechnique Fédérale de Lausanne, Lausanne, Switzerland; 3 Division of Neuroradiology, Department of Medical Imaging, Toronto Western Hospital, University Health Network, Toronto, Ontario, Canada; 4 Division of Neurosurgery, Department of Surgery, Toronto Western Hospital, University Health Network, Toronto, Ontario, Canada; Technion - Israel Institute of Technology, Israel

## Abstract

We investigated the flow modifications induced by a large panel of commercial-off-the-shelf (COTS) intracranial stents in an idealized sidewall intracranial aneurysm (IA). Flow velocities in IA silicone model were assessed with and without stent implantation using particle imaging velocimetry (PIV). The use of the recently developed multi-time-lag method has allowed for uniform and precise measurements of both high and low velocities at IA neck and dome, respectively. Flow modification analysis of both regular (RSs) and flow diverter stents (FDSs) was subsequently correlated with relevant geometrical stent parameters. Flow reduction was found to be highly sensitive to stent porosity variations for regular stents RSs and moderately sensitive for FDSs. Consequently, two distinct IA flow change trends, with velocity reductions up to 50% and 90%, were identified for high-porosity RS and low-porosity FDS, respectively. The intermediate porosity (88%) regular braided stent provided the limit at which the transition in flow change trend occurred with a flow reduction of 84%. This transition occurred with decreasing stent porosity, as the driving force in IA neck changed from shear stress to differential pressure. Therefore, these results suggest that stents with intermediate porosities could possibly provide similar flow change patterns to FDS, favourable to curative thrombogenesis in IAs.

## Introduction

Stent-assisted coiling has been widely used for the treatment of large neck intracranial aneurysms (IAs) [1]. Beside few sole stenting treatments of vertebro-basilar IAs [2], flow diverter stents (FDSs) characterized by low porosity are now extensively used to promote flow reduction and induce progressive thrombosis in IAs. Despite of the successful treatment outcome reported for large and giant IAs in the anterior circulation [3], the use of FDS has been associated with some complications, including subacute aneurysm rupture and parent artery occlusion [4]. Furthermore, incomplete aneurysm thrombosis in the long-term follow-up was also reported. However, the lack of knowledge regarding these issues has prevented the physicians from optimizing their choices of devices out of the large collection of available commercial-off-the-shelf (COTS) stents. Recently, in clinical studies, FDS treatment issue was found correlated with hemodynamic change factors measured per-operatively [5], [6] with digital subtracted angiography (DSA) and predicted numerically [7]–[9] with computational fluid dynamics (CFD). Despite the consistency of such results with low velocity and wall shear stress condition hypothesis in triggering the pathway for currative thrombosis formation [10], [11], the details of the biological event scenario leading to thrombogenesis are still unknown.

In the meanwhile, in-vitro experiments using particle imaging velocimetry (PIV) technique are readily available to measuring the hemodynamic change involved in the sealing process induced by FDS. PIV is an in-vitro technique that assesses velocity fields by measuring the displacement of tracing micro particles embedded within the circulating fluid. Different investigations have been performed in stented IA phantoms using various experimental configurations [12]–[30] with two fold purposes in sidewall IAs: 1/Hemodynamic effect related to stent porosity using prototypes especially designed to get different shapes, and various permeabilities in both idealized IAs [15], [17], [20], [24]–[29] and realistic models [13], [14], [22]; 2/Comparison of a clinically accepted FDS [21] to different number of interlacing layers of regular stents (RSs). In general, flow reduction was shown to correlate with stent porosity and permeability along with strong modification of IA flow pattern. However, in all of those studies, the sensitivity of PIV techniques used were mainly limited to high velocities close to IA neck, far from IA dome where the salient features related to stent moderation usually occur [31]. Consequently, the global understanding of these flow pattern modifications was still lacking, while on the clinical side, these investigations were restricted to a single type of existing FDS or prototypes, ignoring the large number of devices available for patients.

In this study, we investigated IA flow modification induced by a broad panel of COTS stents with high (RS) and low (FDS) porosity to correlate clinically available strut densities with flow modulation effects. Moreover, flow change was assessed using the recently developed multi-time-lag (MTL) PIV method [32], allowing for precise and uniform measurements at both IA neck and aneurysm fundus despite of the large velocity differences (up to three order of magnitude). Stent design factors such as porosity and permeability were also measured and correlated with flow change in stented IAs using appropriate parameters, along with providing a new physical interpretation of the observed events.

## Methods

### IA model

**Figure 1:**
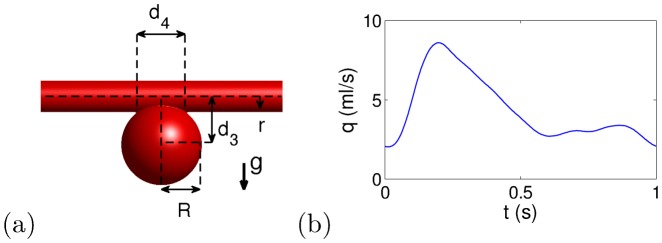
Idealized sidewall IA model geometry in (a). Gravity direction **g** points down to the dome. Time dependence of the inflow rate in (b).

The idealized sidewall IA model, shown in Fig. 1.a, was previously described in Ref. [32]. It is composed of a cylindrical artery with radius *r* = 2 mm and a sphere with radius *R* = 5 mm located at distance *d_3_* = 6 mm below the artery center (neck width *d_4_* = 6 mm and aspect ratio 1.5, i.e. ratio of aneurysm depth to neck width). This simplified geometry ensured: **a**/stent deployment that minimizes strut protrusion and cell deformation, and the geometrical characteristics of each deployed stent are uniform and precisely measured; **b**/investigation of shear and pressure driven IA flow; **c**/relevant IA symmetry plane allowing 2D PIV to be performed with negligible out-of-plane, inaccessible velocity component.

### PIV measurements

**Figure 2:**
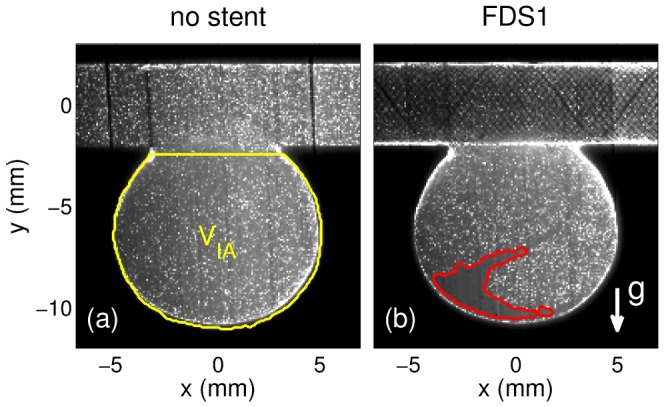
Raw PIV picture of the seeding particles in the idealized IA model without (a) and with (b) FDS1 implanted. The yellow line in (a) defines the IA domain, *V_IA_*. The red line in (b) shows the contour of the free-seeding-particle domain in the IA dome.

The PIV experimental setup has been previously detailed in Ref. [32], as has the MTL acquisition method, which allows for precise and uniform assessment of both high and low velocities at IA neck and dome, respectively. The measurements were performed at the symmetry plane of IA model, focusing on IA domain, *V_IA_* (see Fig. 2), which was determined using a µCT scan of the phantom [32]. The circulating fluid was a mixture of glycerin (59.1%) and water (40.9%), heated at 37°C for ensuring the correct deployment of thermal shape-memory implants with density ρ*_f_* = 1142 kg/m^3^ and kinematic viscosity ν*_f_* = 4.67•10^−6^m^2^/s (blood: ρ*_b_* = 1060 kg/m^3^ and ν*_b_* = (3–4)•10^−6^m^2^/s). The imposed inflow rate, *q*(t), shown in Fig. 1.b had the typical waveform of the blood flow in an internal carotid artery (ICA) [33] corresponding to a period of *T* = 1 s and Reynolds numbers (Re = 2*q*/π*r*ν) ranging between 140 and 590. During this period, 10 equally spaced PIV measurements were performed. To measure velocities after stent implantation, MTL time lags, Δ*t*_α_ [32] were fixed differently for stented IA (Δ*t*_α_  = 2.5, 10 and 40 ms) and for unstented IA (Δ*t*_α_ = 0.1, 0.4, 1.6, and 6.4 ms).

### Stent implantation and characterization

We have investigated the effects of 4 different high-porosity φ (hole surface density) RSs and 3 low-porosity FDSs (see Table. 1) that are in current clinical use. To avoid commercial exploitation of this study, the stent models were arbitrarily named RS1-4 and FDS1-3 and numbered in descending order of porosity. Each stent was delivered and implanted in accordance with clinical practice whereby a 6F introducer sheath was placed in one arm of Y-pipe equiping the inlet of the model, allowing further the insertion of catheters under flow condition in the model without leakage. A double coaxial catheter system (6F guiding catheter associated with the catheter dedicated to the stent) was used to navigate distally from aneurysm region with the help of a 0.014 inch guide wire. Finally, the stent was deployed following the manufacturer's guidelines under live control of the PIV camera.

**Table 1:** List of implanted regular stents (RSs) and flow diverter stents (FDSs).

Type	Model	Company
RS	Neuroform 4/15	Stryker Neurovascular
		Kalamazoo, USA
	Leo 3.5/18	Balt Extrusion
		Montmorency, France
	Acclino 4.5/25	Acandis
		Pforzheim, Germany
	Enterprise 4.5/28	Codman & Shurtleff
		Raynham, USA
FDS	FD 4.5/30	Acandis
		Pforzheim, Germany
	Silk 4/20	Balt Extrusion
		Montmorency, France
	Pipeline 4/14	Covidien
		Irvine, USA

The two numbers following the model name are the nominal stent diameter and length (in mm).

**Figure 3:**
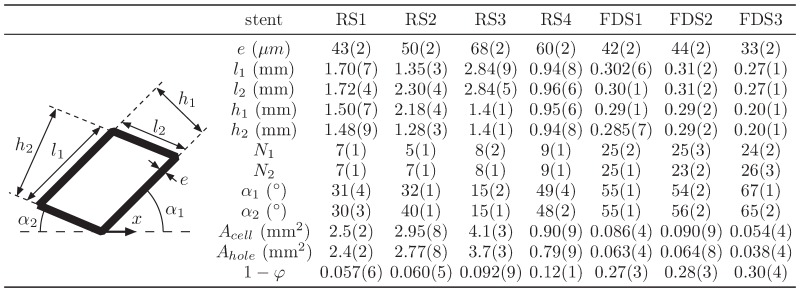
Picture of the assumed parallelogram stent unit cell and table of the stent geometrical dimensions in descending order of porosity, φ = *A_hole_/A_cell_*. *A_cell,hole_* are the unit cell and hole surface, respectively. The index 1, 2 identifies the strut orientation. *x*-axis shows parent artery orientation. is the wire number (assuming a woven stent made of two different wire orientations). The number in parentheses represents the 95% confidence interval on the last digit.

The geometrical characteristics of the stents were subsequently measured assuming a parallelogram stent unit cell (see Fig. 3). Pictures recorded with the PIV camera at the center of the parent artery provided the strut angles α_1,2_ with respect to the parent artery direction (represented by the *x* axis) and unit cell sizes *l_1,2_*. The wire strut *e* was measured using optical microscopy. The inter-strut distance, *h_1,2_*, porosity, φ = *A_hole_/A_cell_*, and surface of the unit cell and hole, *A_cell,hole_*, were all deduced from the primary endpoints. The permeability scaling factor, proposed in Ref. [34], min(*h_i_*)^2^φ, was also computed for further correlation with IA flow reduction.

### Definition of flow parameters

The spatio-temporal characterization of the measured velocity fields requires reduced parameters such as time-averaged velocity, 
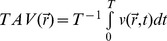
, and oscillation velocity index, 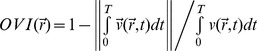, which quantify the local velocity magnitude and flow pattern variations during the cardiac cycle (OVI≈0 when the velocity field direction remains steady during the entire cycle, OVI≈1 for strongly varying flow patterns), respectively. The spatially averaged IA velocity, SAV (velocity averaged over IA domain), quantifies the temporal evolution of IA velocity magnitude. The flow reduction effect of the stent is evaluated with the averaged IA velocity ratio (AVR), which is the ratio of IA velocities (with/without stenting) averaged over one period in IA domain.

## Results

### Stent properties

The measured properties of stents deployed in the same IA model are summarized in Fig. 3. The geometrical properties were measured with only few percent of error (see digits in parenthesis in Fig. 3) showing the high uniformity of the stent deployment and are in agreement with previous studies [35]–[37]. For all stents, the symmetrical deployment of wires in cylinder is such that α_1_≈α_2_ and *l*_1_≈*l*_2_, except for RS2 deployment, in which *l*_2_≈2*l*_1_. The FDS struts tended to be thinner (*e* = 33–44 µm) than those used in RS (*e* = 43–68 µm), and the total number of FDS wires was ≈3 times larger (*N*_1,2_≈24 and *N*_1,2_≈8 for FDS and RS, respectively). Consequently, the FDS metal surface density (1–φ = 0.27–0.3) was more than 3 times larger than in regular stents RS1-3 (1–φ = 0.057–0.092). Similarly, the FDS unit cell area (*A_cell_* = 0.054–0.09 mm^2^) was typically 1–2 orders of magnitude lower than in RS1-3 (*A_cell_* = 2.5–4.1 mm^2^). It is worth noting that RS4 had an intermediate metal surface density (1−φ = 0.12) and unit cell area (*A_cell_* = 0.9 mm^2^) when compared to both RS1-3 and FDS1-3.

### Flow description: influence of stent properties

**Figure 4:**
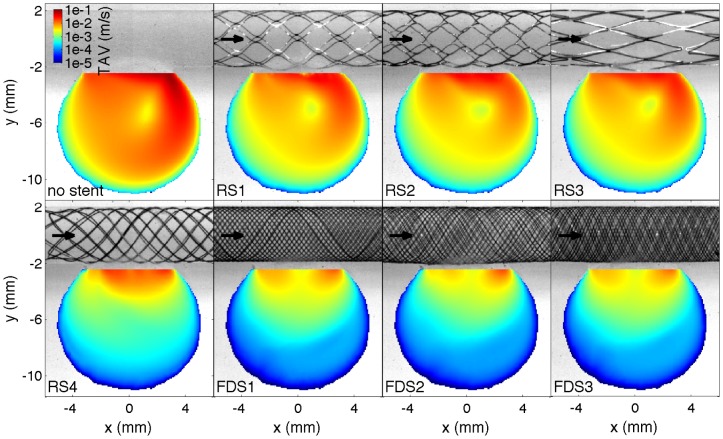
Time averaged velocity map, TAV, in the unstented and stented idealized IA. The black arrows represent the flow direction.

**Figure 5:**
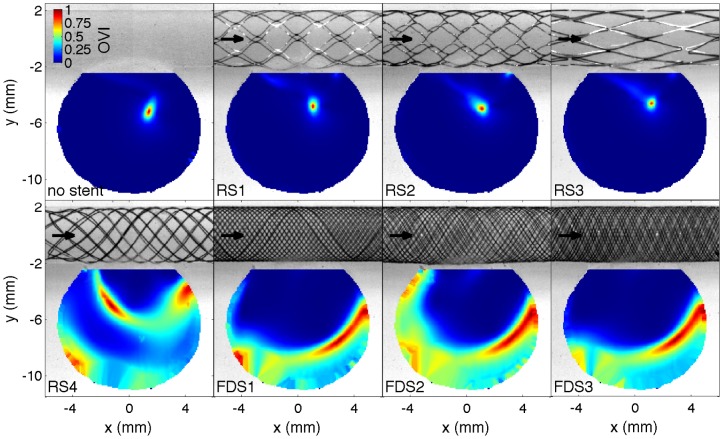
Oscillation velocity index map, OVI, in the unstented and stented idealized IA. The black arrows represent the flow direction.

**Figure 6:**
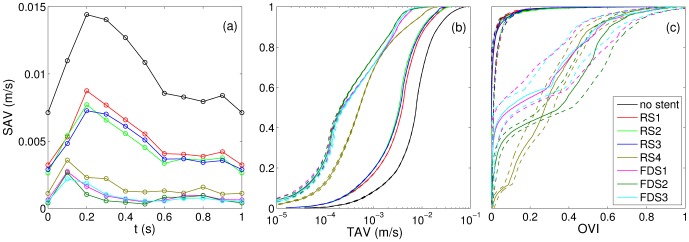
Time dependence of the spatially averaged IA velocity, SAV, in (a). Cumulative distribution of the time averaged IA velocity, TAV, in (b). Cumulative distribution of the oscillation velocity index, OVI, in (c). The dashed lines represent the 95% confidence interval.

**Figure 7:**
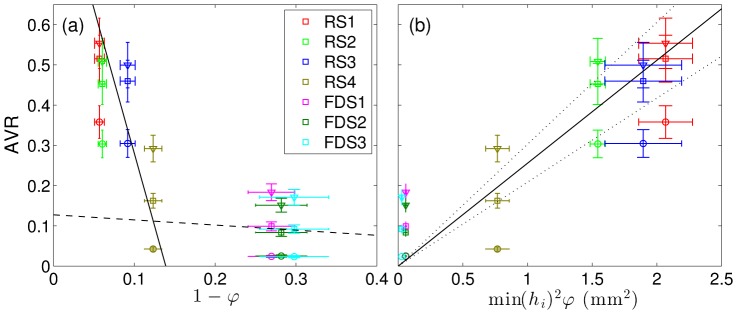
Time-space averaged IA velocity ratio, AVR versus (a) the metal surface density, 1−φ, and (b) the scaling factor of the permeability, min(*h_i_*)^2^φ, proposed in Ref. [34]. The squares, triangles and circles show AVR computed in the full IA domain and in the volumes spanning the highest and lowest 25% of velocities. The black solid and dashed lines represent the linear trends (AVR(φ) = 7.2 φ −6.2 and AVR(φ) = 0.13 φ in (a), respectively). The error bars and dotted line (in (b)) represent 95% confidence intervals.

Figs. 4 and 5 show colormap of the TAV and OVI with and without each stent implantation. The cumulative distribution of both TAV and OVI is also presented in Fig. 6 along with the temporal evolution of the SAV. Fig. 7 summarizes the flow reduction effect of the tested stents. The AVR is plotted against the metal surface density, 1−φ, in Fig. 7.a and permeability scaling factor in Fig. 7.b. A MAT-file (Matlab) (Data S1) and a movie (Movie S1) of the measured velocity fields are also available online.

**Figure 8:**
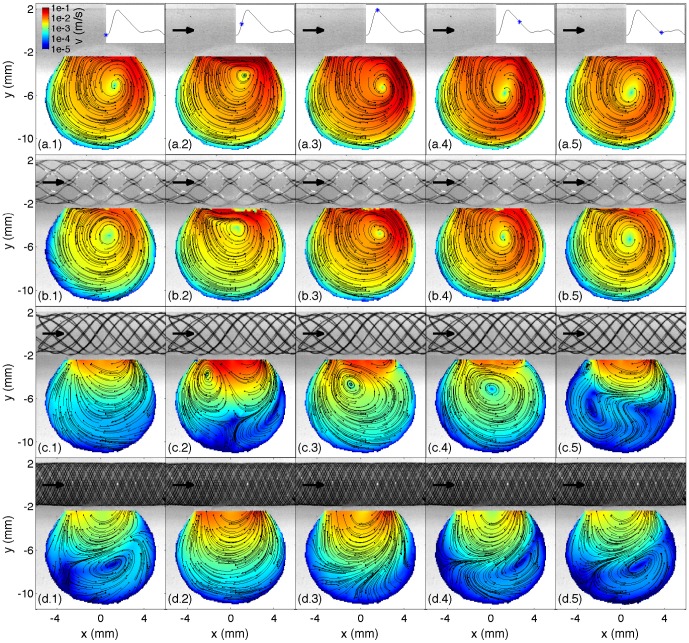
Velocity map, *v*, and planar streamlines measured at time phase *t* = 0, 0.1, 0.2, 0.4 and 0.6 s (in column (1)-(5)) in the symmetry plane of the idealized sidewall IA without stent (row (a)) and with RS1, RS4 and FDS3 (in row (b), (c) and (d)). The time phase is represented by a star in the inflow rate time curve inserted at the top of each column. The black arrows represent the flow direction, and the little black dots indicate the beginning of the streamlines.

The two groups of stents, RS1-3 and FDS1-3, clearly showed flow reductions of ≈50% and ≈90%, respectively, as well as different flow pattern configurations. Within each group, a specific flow reduction trend with regard to stent porosity was observed in Fig. 7.a. The linear decreasing slope in the high-porosity group was 56 times larger than in the low porosity group. Consequently, the stents with high porosities were highly sensitive to porosity variation (black line) and those with lower porosities were weakly sensitive to porosity variation (dashed lines). RS4 with intermediate porosity and permeability values showed an intermediate flow pattern including features of both high- and low-porosity groups. RS4's flow reduction of ≈84% was slightly lower than FDSs ones and was located at the intermediate porosity characterizing high and low flow reduction trends (Fig. 7.a). The comparison of the three relevant flow configurations (i.e. RS1-3, RS4 and FDS1-3) with unstented IA flow is discussed in the following sub-sections and illustrated in Fig. 8. This figure shows their velocity magnitude, *v*, at different time intervals, along with their streamlines through unstented IA (Fig. 8.a), high porosity stent (RS1, Fig. 8.b), intermediate porosity stent (RS4, Fig. 8.c) and low porosity stent (FDS3, Fig. 8.d).

#### RS1-3

Although velocity reduction is strongly sensitive to porosity in the high-porosity group, the low metal surface density of RS1-3 does not fundamentally affect IA flow structure. As detailed in Ref. [32], the flow pattern results from the collision between the main central clockwise swirl induced by a distal inflow jet and a transient secondary vortex that appears at the proximal side of the neck when the inflow accelerates during the early phase of the systole [38], [39]. Due to the small velocity reduction at the neck, the secondary vortex lasts longer before collapsing with the main swirl. Consequently, both TAV and the corresponding cumulative distributions are similar to the ones with unstented IA, after considering appropriate velocity reduction rescaling (≈50%). However, these stents reduce the highest quartile in velocity, close to the neck and in the inflow jet, slightly less than the lowest quartile in velocity located at IA dome (flow reduction of ≈45% and ≈65%, respectively, as shown in Fig. 7). OVI≈0 characterizes the stability of flow pattern in the main part of IA, away from the main vortex core or along the secondary vortex trajectory. The corresponding cumulative distributions match the ones for unstented IA and the time dependence of SAV is similar to the inflow waveform.

#### FDS1-3

Unlike the results observed with RS1-3, velocity reduction is less sensitive to FDS1-3 porosity, and their low-porosity strongly modifies IA flow pattern. As reported previously using similar sidewall IA [17], [20], [22], [24], [26]–[29], IA inflow-outflow is reversed from the proximal side to the distal side of the neck. A low motion eddy is then formed at the dome and shrinks when the inflow rate increases. This eddy is completely washed out at the early phase of systole, inducing a stronger peak in the SAV. As shown in both TAV and OVI maps, the most stable high velocities are concentrated close to the neck. Alternatively, a value of OVI≈1 in low-motion eddy indicates susceptibility to inflow waveform. Consequently, the cumulative distributions for TAV are broader than those in the unstented and RS1-3 cases, with a high concentration (≈50%) of very low velocities (*v*<2•10^−4^m/s) in large eddy area and a low concentration (<3%) of higher velocities (*v*>5•10^−3^m/s) close to the neck. Flow reduction of the lowest quartile in IA velocities (>97%) improves when compared to the highest quartile (≈83%), as shown in Fig. 7. Although OVI cumulative distributions are similar for all FDS1-3, a slightly higher rate of high OVI marks FDS2 compared to both FDS1 and FDS3. These high OVIs are concentrated at the proximal side of the neck due to a transient backflow emerging at peak systole.

#### RS4

The regular stent RS4, with intermediate porosity and permeability, shows transitional flow pattern between the two main regimes discussed above. Although IA inflow-outflow at the neck is inverted with regard to unstented and RS1-3 stented IA, a broad vortex appears proximally during the early phase of systole, which is similar to the secondary vortex reported in unstented IA under pulsatile flow. During systole, this vortex moves slowly across IA before vanishing with a low inflow rate at diastole. This vortex induces an additional backflow at the proximal side of the neck. Similarly to FDS group, the highest quartile in velocity is located at the neck though reduced less by ≈70% compared to FDS (≈83%, see Fig. 7). Moreover, the lowest quartile at IA dome for RS4 is reduced by ≈96% as for FDS. As to TAV, it is slightly shifted compared to the one for FDS, including higher velocities (≈10% of higher velocities with *v*>5•10^−3^m/s). Finally, the strong fluctuations in flow pattern during the cycle induce a broad distribution of OVI with the highest values (≈1) located along the trajectory of the transient vortex and at IA dome.

## Discussion

### Physical interpretation of flow transition

**Figure 9:**
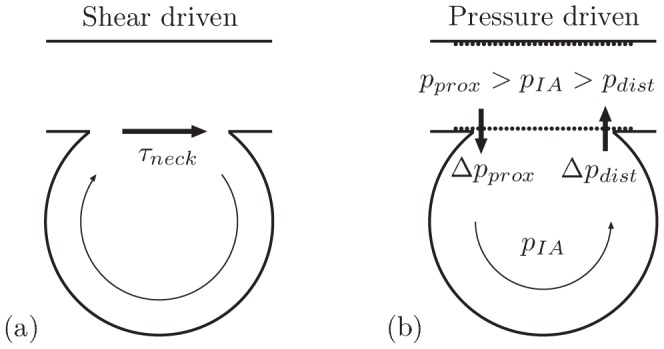
Schematic representation of the two main flow regimes and their physical interpretation. (a) In the shear driven regime, the shear stress at the IA neck, 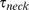, induced by the strong velocity gradient in the parent artery, drives a clockwise intra-aneurysmal flow. (b) In the pressure driven regime, the pressures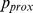 and 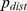 at the proximal and distal side of the neck in the parent artery are such as 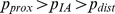 due to the increase of pressure gradient along the parent artery after stent implantation (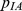 being the IA pressure). Consequently, the pressure differentials between the parent artery and the IA, 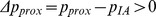 and 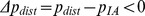, push the circulating fluid inward and outward the IA at the proximal and distal side of the neck, respectively, leading to a counterclockwise flow at the IA neck.

The transition between the two main flow regimes occured when reducing the open space at neck. The experimental conditions are such that, since the inflow inertia is poorly transmitted to IA from straight parent artery, the shear stress exchanged at neck drives the unstented IA flow. After stent implantation, the friction on the struts, which was decreasing the transmission of shear stress through the neck, is counteracted by an increase of adverse pressure gradient along the parent artery. Unlike for RS1-3, shear stress transmission is very low for FDS1-3. In the latter, the pressure gradient along the parent artery leads to positive/negative pressure differential through the stent at the proximal/distal side of the neck. As described in Fig. 9, this pressure differential pushes the circulating fluid inward/outward the IA at the proximal/distal side of the neck inducing the observed pressure driven flow pattern for FDS1-3. The transition between the shear and pressure driven regimes results in much greater flow reduction, particularly to low-velocity zones, due to the formation of a large, almost stagnant eddy with low shear rate that promotes thrombogenesis [40]. This flow domain separation pattern (circulation-stagnation) has been observed clinically using digital subtracted angiography (DSA) [41], [42]. Similar hemodynamic transition has also been predicted with computational fluid dynamics (CFD) after virtual FDS implantation in successfully treated patient specific sidewall IAs geometries [9].

Flow reduction after stent implantation is known to promote the thrombus formation that leads to IA exclusion and progressive vascular remodeling [8]. In patient, the flow field evolves while the thrombus forms modifying the circulating volume. Despite various attempts to model thrombosis [11], [43], the difficulty in precisely determining IA flow in vivo has prevented these models from being confirmed clinically.

Lacking a precise hemodynamic factor to trigger thrombosis, the present trend in FDS design is to increase the number of wires, which drives faster aneurysm exclusion. However, the risk of side branch occlusion [21] and the rigidity of the prosthesis both increase with the density of wires, while the stent porosity is lowered; both effects can cause medical complications.

Producing flow reduction comparable to FDS1-3, RS4 would reduce the risk of vessel perforator obstruction and improve navigability due to its lower wire density. This suggests that hemodynamics prone to thrombogenesis could be achieved with a low wire density FDS if the transition to a low velocity hemodynamic regime could occur, as was already reported in patients by Zenteno et al. [2], who observed spontaneous thrombosis in IA treated with RS.

To confirm this promising result, additional investigations on patient specific geometries are therefore strongly required to include the effect of inflow inertia transmission.

### Stent and model geometries

Although comparable flow reductions have been measured in previous studies with similar straight sidewall IA and stent porosities, the results of those studies showed high sensitivity to IA model and stent geometries. For instance, IA flow reductions of ≈50% and ≈80% were estimated for high (87%) and low (63%) stent porosity in Ref. [15]. A lower IA flow reduction (≈60%) was measured in Ref. [24] after helix and mesh stent implantation with 70% porosity. In contrast, flow reduction of 94–98% using stents with porosities ranging from 80% to 64% was reported in Ref. [29]. In models using IA located on a curved parent artery or a bifurcation, the reported flow reduction was generally much lower [18], [19], [27] due to increased inertia.

To focus on clinical relevance, we used commercially available devices with their inherent geometry and porosity. The influence of the stent pore size was investigated in Refs. [26], [27] by tuning the wire width while keeping porosity constant using spring [26] and mesh [25] prototype geometries. Both studies reported IA flow reduction corresponding to decreasing pore size due to viscous force increase through the struts. Although this effect could not be tested in the present study due to the limited number of available stents, we expect a similar effect especially around RS4 porosity, where the transition between the two flow regimes occurs.

In Ref. [34], the theory of porous media [44] was used to model flow though a mesh of perpendicularly crossing cylinders. The permeability *k*, linking the flow rate through the square mesh with the pressure gradient, was reportedly scaled with 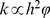 (*h* being the inter-wire distance of the square strut). As opposed to the porosity, φ, the permeability takes into account the geometrical properties of the stent. For the parallelogram struts in Fig. 3, with inter-wire distance, *h_1,2_*, the scaling factor min(*h_i_*)^2^φ is correlated with AVR in Fig. 7.b. For high-porosity stents RS1-4, the stronger flow reduction caused by RS4 in comparison to RS1-3 seems to scale rather well with its lower permeability. In contrast, the low-porosity FDS1-3 correspond to lower flow reduction than predicted by the scaling theory. These different trends are analogous to those observed in response to changes in stent porosity. Based on the Darcy law limited to steady low velocities, this model should be extended to take into account the large velocities in the parent artery and the flow angle.

The effect of inhomogeneity in array design was also investigated in Ref. [34] with results indicating that permeability increases with disorder. In the present study, the lower uniformity of the FDS2 mesh, compared to FDS1 and FDS3 mesh, could have led to the transient backflow observed at the proximal side of the neck (see the larger OVI in Fig. 5). As shown in Fig. 3 and observed in Fig. 5, FDS2 has ≈2 times larger relative uncertainties on the strut sizes *l_1,2_* than for FDS1,3. Stent uniformity is therefore important because it avoids undesired flow jets and minimizes the positioning effect.

### Measurement limitations

The PIV measurements focused on the symmetry plane of IA, which captures the main IA flow features but does not show the 3D nature of the velocity field [45], [46], hence requiring additional investigations.

The longer delays used between consecutive pictures of stented IA caused time averaging biases [47], which were partially corrected by rescaling velocities in the different time-lag domains such that the difference at their boundary layers was minimized. Nevertheless, the very low velocities at IA dome could not be properly measured due to the buoyancy effect preventing the seeding particles to reach such part of IA dome (see Fig. 2.b). A non-slip boundary condition was used to interpolate the velocities in the empty-particle domain as well as near the entire IA wall (width ≈0.25 mm).

## Conclusions

This study investigated the hemodynamic impact of intracranial stents in IA using multi-time-lag PIV. In models using currently available stents, two distinct flow patterns were identified using high (RS1-3)- and low (FDS1-3)-porosity COTS stents, which showed velocity reductions of up to 50% and 90%, respectively. Velocity reduction was strongly sensitive to RS1-3 porosity and was slightly sensitive to FDS1-3 porosity. Any hypothetically lower FDS porosity would not much improve the therapeutic flow change effect. We claim that these distinct flow patterns are the result of different driving forces in the idealized IA flow (shear stress or differential pressure). A transitional regime was observed when using a stent with intermediate porosity (RS4), for which a velocity reduction of 84% was observed. These results suggest that stents with intermediate porosities could still provide the same flow change patterns as FDS. This result may lead to new low wire density FDS designs if confirmed with patient specific geometries, further.

## Supporting Information

Data S1 Measured velocity fields in MAT-file (Matlab) format.
(MAT)

Movie S1 Time evolution of the velocity map, *v*, and planar streamlines measured in the symmetry plane of the IA without stent and with RS1-4 and FDS1-3.
(AVI)
